# Correction to “A Case of Stevens–Johnson Syndrome Triggered by Methicillin‐Resistant *Staphylococcus aureus* Infection During Pembrolizumab Treatment for Lung Squamous Cell Carcinoma”

**DOI:** 10.1002/rcr2.70318

**Published:** 2025-08-19

Y. Irie , K. Isobe , R. Ohash , K. Namba , M. Iwayanagi , H. Sakurai , D. Sakai , K. Takashima , Y. Murakami , K. Kaneko , S. Mitsuyama , H. Wakabayashi , Y. Matsuzawa , “A Case of Stevens–Johnson Syndrome Triggered by Methicillin‐Resistant *Staphylococcus aureus* Infection During Pembrolizumab Treatment for Lung Squamous Cell Carcinoma,” Respirology Case Reports 13, no. 7 (2025): e70290, 10.1002/rcr2.70290.40704213 PMC12283215

The name of the eleventh author “Nobuharu Mitsuyama” was incorrect. This should be corrected to “Shinji Mitsuyama.”

The article online has been corrected as well.

We apologize for this error.

